# Supplementary material for: Biological Actions of Alamandine: A Scoping Review
Source: Biomedicines. 2025 Aug 11;13(8):1957. doi: 10.3390/biomedicines13081957 (PMC12383702; doi:10.3390/biomedicines13081957)
Supplement: Supplementary file 1 [file biomedicines-13-01957-s001.zip › biomedicines-3774550-supplementary.pdf]

biomedicines

MDPI

## Supplementary Materials

**Supplementary Table S1**. Results of PubMed, Scopus, Embase and Web of Sciences search. Last search: 01.30.2025

| | |
|---|---|
| Pubmed (n = 141) | (((((((Alamandine[Title/Abstract])) OR (Ala-Arg-Val-Tyr-Ile-His-Pro[Title/Abstract])) OR (MRGPRD protein, human[MeSH Terms])) OR (MRGPRD protein, human[Title/Abstract])) OR (Mas-related G-protein-coupled receptor, member D, human[Title/Abstract])) OR (MAS related GPR family member D protein, human[Title/Abstract])) OR (MrgD protein, human[Title/Abstract]) |
| Scopus (n = 153) | ( TITLE-ABS-KEY ( "Alamandine" ) OR TITLE-ABS-KEY ( "Ala-Arg-Val-Tyr-Ile-His-Pro" ) OR TITLE-ABS-KEY ( "MRGPRD protein, human" ) OR TITLE-ABS-KEY ( "Mas-related G-protein-coupled receptor, member D, human" ) OR TITLE-ABS-KEY ( "MAS related GPR family member D protein, human" ) OR TITLE-ABS-KEY ( "MrgD protein, human" )) |
| Embase (n = 149) | 'Alamandine ':ab,ti OR ' Ala-arg-val-tyr-ile-his-pro':ab,ti OR 'mrgprd protein, human':ab,ti OR 'mas-related g-protein-coupled receptor, member d, human':ab,ti OR 'mas related gpr family member d protein, human':ab,ti OR 'mrgd protein, human':ab,ti |
| Web of Science (n = 147) | Alamandine* (Tópico) or "Ala-Arg-Val-Tyr-Ile-His-Pro" (Tópico) or "MRGPRD protein, human" (Tópico) or "Mas-related G-protein-coupled receptor, member D, human" (Tópico) or "MAS related GPR family member D protein, human" (Tópico) or "MrgD protein, human" (Tópico) |

**Supplementary Table S2** - Summary of data extraction.

| |
|---|
| Publication information: date, journal title, language, and impact factor. |
| Study design: experimental. |
| Location: Pathology / Body system involved |
| Population: In Vivo, In Vitro, In Silico |
| Main discoveries: Mechanism of action and effects of Alamandine |

 https://doi.org/ 10.3390/biomedicines13081957

**Supplementary Table S3** – Preferred Reporting Items for Systematic reviews and Meta-Analyses extension for Scoping Reviews (PRISMA-ScR) Checklist

| SECTION | ITEM | PRISMA-ScR CHECKLIST ITEM | REPORTED ON PAGE # |
|---|---|---|---|
| TITLE | | | |
| Title | 1 | Identify the report as a scoping review. | Yes. Page 1 |
| ABSTRACT | | | |
| Structured summary | 2 | Provide a structured summary that includes (as applicable): background, objectives, eligibility criteria, sources of evidence, charting methods, results, and conclusions that relate to the review questions and objectives. | Structured summary provided above. Page 1 |
| INTRODUCTION | | | |
| Rationale | 3 | Describe the rationale for the review in the context of what is already known. Explain why the review questions/objectives lend themselves to a scoping review approach. | The review aims to map mechanisms of action of ALA, an area with clear knowledge gaps. Page 1–2 (introductory paragraphs) |
| Objectives | 4 | Provide an explicit statement of the questions and objectives being addressed with reference to their key elements (e.g., population or participants, concepts, and context) or other relevant key elements used to conceptualize the review questions and/or objectives. | ALA: What mechanisms of action are described? Subquestions: Under which clinical conditions? What effects? Page 2 (Section 2.1) |
| METHODS | | | |
| Protocol and registration | 5 | Indicate whether a review protocol exists; state if and where it can be accessed (e.g., a Web address); and if available, provide registration information, including the registration number. | Yes. Page 2 (Section 2, registered with OSF: DOI 10.17605/OSF.IO/CG9U4) |
| Eligibility criteria | 6 | Specify characteristics of the sources of evidence used as eligibility criteria (e.g., years considered, language, and publication status), and provide a rationale. | No date/language restrictions. Excluded: abstracts, commentaries, reviews, editorials, conferences. Page 2 (Section 2.2) |
| Information sources* | 7 | Describe all information sources in the search (e.g., databases with dates of coverage and contact with authors to identify additional sources), as well as the date the most recent search was executed. | Page 2 (Section 2.2 – Databases: PubMed, Embase, Scopus, Web of Science; Updated on 01/30/2025) |
| Search | 8 | Present the full electronic search strategy for at least 1 database, including any limits used, such that it could be repeated. | Suplementary Table 1 |
| Selection of sources of evidence† | 9 | State the process for selecting sources of evidence (i.e., screening and eligibility) included in the scoping review. | Review in Rayyan® by two independent reviewers (ATS and JF); discrepancies resolved by consensus. Page 2–3 (Section 2.3) |
| Data charting process‡ | 10 | Describe the methods of charting data from the included sources of evidence (e.g., calibrated forms or forms that have been tested by the team before their use, and whether data charting was done independently or in duplicate) and any processes for obtaining and confirming data from investigators. | Excel extraction by ATS and JF; pilot test with 5 articles. Page 3 (Section 2.4) |

| SECTION | ITEM | PRISMA-ScR CHECKLIST ITEM | REPORTED ON PAGE # |
|---|---|---|---|
| Data items | 11 | List and define all variables for which data were sought and any assumptions and simplifications made. | Body system, experimental model, molecular pathways, biological effects (anti-inflammatory, antifibrotic, etc.). Supplementary Table 2 |
| Critical appraisal of individual sources of evidence§ | 12 | If done, provide a rationale for conducting a critical appraisal of included sources of evidence; describe the methods used and how this information was used in any data synthesis (if appropriate). | No formal critical evaluation was carried out, given the scope of the review. |
| Synthesis of results | 13 | Describe the methods of handling and summarizing the data that were charted. | Descriptive statistics; tables and graphs to present data. Page 3 (Section 2.5) |
| RESULTS | | | |
| Selection of sources of evidence | 14 | Give numbers of sources of evidence screened, assessed for eligibility, and included in the review, with reasons for exclusions at each stage, ideally using a flow diagram. | 590 identified, 26 included; reasons for exclusion: n=41 Page 3 (Section 3.1, Figure 1) |
| Characteristics of sources of evidence | 15 | For each source of evidence, present characteristics for which data were charted and provide the citations. | Supplementary Table 4; majority of studies from 2022; all experimental. Page 3 (Section 3.1), Supplementary Table 4 |
| Critical appraisal within sources of evidence | 16 | If done, present data on critical appraisal of included sources of evidence (see item 12). | Not applicable. |
| Results of individual sources of evidence | 17 | For each included source of evidence, present the relevant data that were charted that relate to the review questions and objectives. | Detailed in Supplementary Table 4; effects on cardiovascular, renal, pulmonary systems, etc. Pages 4–13 (Sections 4.1–4.5) |
| Synthesis of results | 18 | Summarize and/or present the charting results as they relate to the review questions and objectives. | ALA has anti-inflammatory, antifibrotic, vasodilatory, and antioxidant effects via MAPK, AMPK, NO, PI3K/Akt. Pages 13–15 (Discussion and Conclusion) |
| DISCUSSION | | | |
| Summary of evidence | 19 | Summarize the main results (including an overview of concepts, themes, and types of evidence available), link to the review questions and objectives, and consider the relevance to key groups. | ALA shows promise in cardiovascular conditions, fibrosis, cancer. It acts via multiple signaling pathways. Pages 13–15 (Discussion and Conclusion) |
| Limitations | 20 | Discuss the limitations of the scoping review process. | Limitation in the number of studies and complete understanding of the mechanisms. Page 15 (last paragraph of the Conclusion) |
| Conclusions | 21 | Provide a general interpretation of the results with respect to the review questions and objectives, as well as potential implications and/or next steps. | ALA has great therapeutic potential. Further studies are recommended, especially in humans. Page 15 (Conclusion) |

| SECTION | ITEM | PRISMA-ScR CHECKLIST ITEM | REPORTED ON PAGE # |
|---|---|---|---|
| FUNDING | | | |
| Funding | 22 | Describe sources of funding for the included sources of evidence, as well as sources of funding for the scoping review. Describe the role of the funders of the scoping review. | Not applicable. |

JBI = Joanna Briggs Institute; PRISMA-ScR = Preferred Reporting Items for Systematic reviews and Meta-Analyses extension for Scoping Reviews.

* Where sources of evidence (see second footnote) are compiled from, such as bibliographic databases, social media platforms, and Web sites.

† A more inclusive/heterogeneous term used to account for the different types of evidence or data sources (e.g., quantitative and/or qualitative research, expert opinion, and policy documents) that may be eligible in a scoping review as opposed to only studies. This is not to be confused with information sources (see first footnote).

‡ The frameworks by Arksey and O'Malley (6) and Levac and colleagues (7) and the JBI guidance (4, 5) refer to the process of data extraction in a scoping review as data charting.

§ The process of systematically examining research evidence to assess its validity, results, and relevance before using it to inform a decision. This term is used for items 12 and 19 instead of "risk of bias" (which is more applicable to systematic reviews of interventions) to include and acknowledge the various sources of evidence that may be used in a scoping review (e.g., quantitative and/or qualitative research, expert opinion, and policy document).

From: Tricco AC, Lillie E, Zarin W, O'Brien KK, Colquhoun H, Levac D, et al. PRISMA Extension for Scoping Reviews (PRISMAScR): Checklist and Explanation. Ann Intern Med. 2018;169:467–473. doi: 10.7326/M18-0850.

**Supplementary Table S4 -** References and reasons for exclusion of articles during the full-text reading phase of eligibility confirmation (n = 42).

| Reference | Reason for exclusion |
|---|---|
| 1.Almeida JF. Alamandine Improves Cardiac Post-Ischemic Function in Isolated Hearts of TGR (mREN2)27. Hypertension. 2016 | Poster session |
| 2. Jesus IC. Alamandine signaling in cardiomyocytes in health and disease. Hypertension. 2015 | Poster session |
| 3. Leão N. Alamandine-induced vasorelaxation is selectively increased in Sp-SHR. Hypertension. 2016 | Poster session |
| 4. Wilson BA. An angiotensin-(1-7) peptidase in the kidney cortex, proximal tubules, and human HK-2 epithelial cells that is distinct from insulin-degrading enzyme. American Journal of Physiology. Renal Physiology. 2015 | Does not answer the guiding question |
| 5. Canta G. AT1R Blockade Increases the Depressor Effect of Alamandine in Normotensive SD Rats. Hypertension. 2016 | Poster session |
| 6. Sampaio WO. Differences in angiotensin-(1-7)/Alamandine mediated signaling in tumoral and normal cell lines. Hypertension. 2016 | Poster session |
| 7. Passos-Silva DG. Differences in renin-angiotensin system components expression in tumoral and normal cell lines. Hypertension. 2016 | Poster session |
| 8. Oliveira AC. Mas-related G-protein coupled receptor D deficiency leads to a marked dilated cardiomyopathy in mice. Hypertension. 2017 | Poster session |
| 9. Oliveira AC. MRGD expression in cardiovascular related areas. Hypertension. 2015 | Poster session |
| 10. Pawlik WW. Role of Vasoactive Renin-Angiotensin Metabolite Angiotensin1-7 in Intestinal Protection Against Lesions Induced by Ischemia-Reperfusion. Gastroenterology. 2019 | Poster session |
| 11. Tanriverdi LH. Activation of the Mas receptors by AVE0991 and MrgD receptor using Alamandine to limit the deleterious effects of Ang II-induced hypertension. Fundamental & clinical pharmacology. 2023 | Does not answer the guiding question |

| | |
|---|---|
| 12. Stoyell-Conti FF. Pyridoxal 5 Phosphate Decarboxylates Angiotensin II: Putative Mechanism for Generation of Angiotensin A. The FASEB Journal. 2020 | Abstract |
| 13. Stein AC. Are Angiotensin - (1-7) and Alamandine Biomarkers of Heart Ischemia?. Circulation. 2019 | Poster session |
| 14. Matsoukas JN. Diminazene Aceturate Reduces Angiotensin II Constriction and Interacts with the Spike Protein of Severe Acute Respiratory Syndrome Coronavirus 2. Biomedicines. 2022 | Does not answer the guiding question |
| 15. Fernandes RS. Assessment of Alamandine in Pulmonary Fibrosis and Respiratory Mechanics in Rodents. Journal Renin-Angiotensin-Aldosterone System. 2021 | Does not answer the guiding question |
| 16. Gong J. Superoxide anions mediate the effects of angiotensin (1-7) analog, Alamandine, on blood pressure and sympathetic activity in the paraventricular nucleus. Peptides. 2019 | Does not answer the guiding question |
| 17. A new method for measurement of ACE2 activity in tissues using fluorescent angiotensin peptides | Does not answer the guiding question |
| 18. Alamandine but not angiotensin-(1-7) produces cardiovascular effects at the rostral insular cortex | Does not answer the guiding question |
| 19. Decarboxylation of Ang-(1–7) to Ala1-Ang-(1–7) leads to significant changes in pharmacodynamics | Does not answer the guiding question |
| 20. Alamandine Improves Cardiac Post-Ischemic Function in Isolated Hearts of TGR(mREN2)27 | Poster session |
| 21. Alamandine reduces eosinophilic inflammation in an experimental model of asthma | Poster session |
| 22. Alamandine-induced vasorelaxation is selectively increased in Sp-SHR | Poster session |
| 23. An ACE2/Mas-related receptor MrgE axis in dopaminergic neuron mitochondria | Does not answer the guiding question |
| 24. Brown adipose tissue transcriptome unveils an important role of the Beta-Alanine/Alamandine receptor, MrgD, in metabolism | Does not answer the guiding question |
| 25. Discovery and characterization of Alamandine: a novel component of the renin-angiotensin system | Does not answer the guiding question |

| | |
|---|---|
| 26. Effects ofAlamandineon hippocampal slices subject to oxygen and glucose deprivation | Does not answer the guiding question |
| 27. Genetic deletion of the Alamandine receptor mrgd leads to dilated cardiomyopathy in mice | Does not answer the guiding question |
| 28. Kidney Size, Renal Function, Ang (Angiotensin) Peptides, and Blood Pressure in Young Adults Born Preterm: The HAPI Study | Does not answer the guiding question |
| 29. Loss of Angiotensin-Converting Enzyme 2 Exacerbates Diabetic Retinopathy by Promoting Bone Marrow Dysfunction | Does not answer the guiding question |
| 30. Mas-related G protein-coupled receptor D is involved in modulation of murine gastrointestinal motility | Does not answer the guiding question |
| 31. Mesoporous silica nanoparticles loaded with Alamandine as a potential new therapy against cancer | Does not answer the guiding question |
| 32. Activation pattern of ACE2/Ang-(1-7) and ACE/Ang II pathway in course of heart failure assessed by multiparametric MRI in vivo in Tg$\alpha$q*44 mice | Does not answer the guiding question |
| 33. Reduction of angiotensin A and Alamandine vasoactivity in the rabbit model of atherogenesis: differential effects of Alamandine and Ang(1-7) | Does not answer the guiding question |
| 34. Reshaping the Preterm Heart: Shifting Cardiac Renin-Angiotensin System Towards Cardioprotection in Rats Exposed to Neonatal High-Oxygen Stress | Does not answer the guiding question |
| 35. The Effects of Alamandine on the NHE3 Exchanger in in vivo Proximal Tubule of Spontaneously Hypertensive Rats | Abstract |
| 36. Alamandine through MrgD receptor induces antidepressant-like effect in transgenic rats with low brain angiotensinogen | Does not answer the guiding question |
| 37. Differences in cardiovascular responses to alamandine in two-kidney, one clip hypertensive and normotensive rats | Does not answer the guiding question |
| 38. Hypotensive effect induced by microinjection of Alamandine, a derivative of angiotensin-(1-7), into caudal ventrolateral medulla of 2K1C hypertensive rats. | Does not answer the guiding question |

| | |
|---|---|
| 39. The Expression of Alamandine Receptor MrgD in Clear Cell Renal Cell Carcinoma Is Associated with aWorse Prognosis and Unfavorable Response to Antiangiogenic Therapy | Does not answer the guiding question |
| 40. Transdermal Delivery of AT1 Receptor Antagonists Reduce Blood Pressure and Reveal a Vasodilatory Effect on Kidney Blood Vessels. | Does not answer the guiding question |
| 41. Alamandine, a protective component of the renin-angiotensin system, reduces cellular proliferation and interleukin-6 secretion in human macrophages through MasR-MrgDR heteromerization. | Does not answer the guiding question |
| 42. Alamandine attenuates hypertension and cardiac hypertrophy in hypertensive rats. | Does not answer the guiding question |

**Supplementary Table S5 -** Extraction of data from selected articles

| ARTICLE | SPECIES | MECHANISM | BODY SYSTEM | EFFECTS OF ALAMANDINE | REF |
|---|---|---|---|---|---|
| Alamandine acts via MrgD to induce AMPK/NO activation against Ang II hypertrophy in cardiomyocytes. | Cardiomyocytes mice - C57BL/6 and rat cardiomyocytes - NRCMs | Via MrgD induces AMPK/NO | Cardiovascular - cardiac | Antihypertrophic and vasodilator. | [8] |
| Alamandine attenuates sepsis-associated cardiac dysfunction via inhibiting MAPKs signaling pathways | Cardiomyocytes mice - C57BL/6 and Primary cardiomyocytes Sprague-Dawley rats | Via inhibiting MAPK | Cardiovascular - cardiac | Anti-inflammatory, anti-apoptotic ↓cardiac dysfunction ↑cardiac contractility | [6] |
| Alamandine alleviated heart failure and fibrosis in myocardial infarction mice. | Sprague-Dawley rats - Primary cells | Inhibition of oxidative stress | Cardiovascular - cardiac | ↓ Cardiac dysfunction ↓cardiac fibrosis | [9] |
| Alamandine attenuates long-term hypertension-induced cardiac fibrosis independent of blood pressure. | Wistar-Kyoto Rats/SHRs Rats - Primary Fibroblasts (Wistar-Kyoto Rats) | Akt pathway inhibition | Cardiovascular - cardiac | Antifibrotic and antiproliferative | [30] |
| Alamandine improves cardiac remodeling induced by transverse aortic constriction in mice. | C57BL/6J mice | ↓ERK1/2 - ↑AMPK | Cardiovascular - cardiac | ↓Cardiac remodeling (↓MMP-2, ↓TGFβ) | [26] |
| Alamandine significantly reduces doxorubicin-induced cardiotoxicity in rats | Sprague-Dawley rats | ↓caspase-3 activity and apoptosis | Cardiovascular - cardiac | Antioxidant, anti-inflammatory and antiapoptotic | [19] |

| | | | | | |
|---|---|---|---|---|---|
| Alamandine enhances cardiomyocyte contractility in hypertensive rats through a nitric oxide-dependent activation of CaMKII | Sprague-Dawley rats/TGR heterozygous rats (mREN2)27 | NO/CaMKII pathway | Cardiovascular - cardiac | ↓Ca2+ ↑NO ↑Contractility and relaxation of cardiomyocytes | [21] |
| An ACE2-Alamandine Axis Modulates the Cardiac Performance of the Goldfish Carassius auratus via the NOS/NO System. | Teleost fish - Carassius auratus | Via NOS/NO ↓AMPK E Akt | Cardiovascular - cardiac | ↑Contractility ↑stroke volume ↑stroke work ↑cardiac output | [16] |
| Alamandine reverses hyperhomocysteinemia-induced vascular dysfunction via PKA-dependent mechanisms | New Zealand breed rabbits | Via PKA | Cardiovascular - Vascular | Vasodilator | [24] |
| Alamandine attenuates angiotensin II-induced vascular fibrosis via inhibiting p38 MAPK pathway. | C10BL57/J mice - Vascular smooth muscle cells (VSMCs) - Sprague-Dawley rats | Blocking p-p38 expression | Cardiovascular - vascular | Anti-inflammatory and antifibrotic | [32] |
| Alamandine, a derivative of angiotensin-(1-7), alleviates sepsis-associated renal inflammation and apoptosis by inhibiting the PI3K/Ak and MAPK pathways | C10bl57/J mice - human proximal tubular epithelial cell line HK-2 | Inhibition of the PI3K/Akt and MAPK and Fox01 pathway | Renal | Anti-inflammatory | [20] |
| Alamandine protects rat from myocardial ischemia-reperfusion injury by activating JNK and inhibiting NF-κB. | Sprague-Dawley rats | Activation of JNK phosphorylation and inhibition of the NF-κB | Renal | Anti-inflammatory | [27] |

| | | | | | |
|---|---|---|---|---|---|
| | | signaling pathway. | | | |
| Alamandine protects against renal ischaemia-reperfusion injury in rats via inhibiting oxidative stress | Sprague-Dawley rats/ NRK52E rat kidney proximal tubular epithelial cells | Via inhibition of oxidative stress | Renal | Anti-inflammatory | [34] |
| Alamandine alleviates hypertension and renal damage via oxidative-stress attenuation in Dahl rats | Dahl salt-sensitive (SS)/ Dahl salt-resistant (SR)/ HK-2 cells | Inhibition of the PKC/reactive oxygen species signaling pathway | Renal + Vascular | ↓Hypertension, renal dysfunction, renal fibrosis and renal apoptosis | [18] |
| Alamandine/MrgD axis prevents TGF-β1-mediated fibroblast activation via regulation of aerobic glycolysis and mitophagy. | C57BL/6 Mice Primary Cells | ALA/MrgD inhibits TGF-β1-induced LF activation by neutralizing glycolysis via activation of Parkin/LC3-mediated mitophagy | Metabolic | Enhanced TGF-β1-mediated activation of lung fibroblasts to repress glycolysis through downregulation of HK2 and PFKFB3 expression. | [31] |
| Alamandine reduces leptin expression through the c-Src/p38 MAP kinase pathway in adipose tissue | Wistar Mouse Rats - 3T3L-1 adipocyte cells | c-Src/p38 MAP kinase pathway in adipose tissue | Metabolic - Fat | ↓Expression of leptin mRNA and leptin secretion in TA and isolated adipocytes | [29] |
| Phosphoproteomic studies of alamandine signaling in CHO-MrgD and human pancreatic carcinoma cells: | HaCat, CHO, CHO-MrgD, Mia Paca-2 and A549 cells | Inhibition of the BRAF/MKK/ERK and PI3K/AKT pathways and | Metabolic - tumor cells | ↓ Proliferation of two tumor cell lines Mia PaCa-2 and A549 | [13] |

| | | | | | |
|---|---|---|---|---|---|
| An antiproliferative effect is unveiled | | activation of FoxO1 | | | |
| Alamandine Induces Neuroprotection in Ischemic Stroke Models | Sprague-Dawley (SD)/ C57/Bl6 Mice | Attenuation of SOD, catalase and GSH activities | CNS - neurological disorders | Anti-inflammatory e antioxidant | [17] |
| Alamandine injected into the paraventricular nucleus increases blood pressure and sympathetic activation in spontaneously hypertensive rats | Wistar-Kyoto rats/SHRs | cAMP-PKA pathway | CNS - Pressure control | ↑Blood pressure ↑Sympathetic output | [25] |
| Alamandine, a new member of the renin-angiotensin system (RAS), attenuates collagen-induced arthritis in mice via inhibiting cytokine secretion in synovial fibroblasts | DBA/1 J and MH7A mice (cells. Human fibroblasts)/ Primary Rheumatoid Arthritis Fibroblasts (RA-FLS) | Inhibition of the MAPK signaling pathway | Rheumatological - Arthritis | Anti-inflammatory | [15] |
| Alamandine via MrgD receptor attenuates pulmonary fibrosis via nox4 and autophagy pathway | C57B/6 Mice/Primary Fibroblast Lungs | Inhibition of oxidative damage and autophagy induction | Pulmonary | Antifibrotic | [22] |
| Expression and Function of Mas-Related G Protein-Coupled Receptor D and Its Ligand Alamandine in Retina | C57B1/6J wild-type mice, MrgD knockout mice, SD mice, Muller glial cells and ARPE-19 | Attenuated NF-kB | Oftalmologic | ↓ NF-κB, the gene expression of inflammatory cytokines and the production of ROS | [33] |

| | | | | | |
|---|---|---|---|---|---|
| | | | | | |
| Alamandine attenuates oxidative stress in the right carotid following transverse aortic constriction in mice | C57BL/6 mice | Attenuates oxidative stress | Cardiovascular | Antioxidant | [14] |
| Alamandine treatment prevents LPS-induced acute renal and systemic dysfunction with multi-organ injury in rats via inhibiting iNOS expression | Sprague-Dawley rats | via inhibiting iNOS | Renal | Anti-inflammatory, antipyrotic and antiapoptotic | [28] |
| Alamandine attenuates ovariectomy-induced osteoporosis by promoting osteogenic differentiation via AMPK / eNOS axis | Sprague-Dawley rats/ The mouse embryo osteoblast precursor (MC3T3-E1, subclone 14) cell line | AMPK/eNOS | Musculoskeletal | attenuate the postmenopausal osteoporosis progression | [23] |
